# [1,3-Bis(2-ethoxy­phen­yl)triazenido]chloridomercury(II)

**DOI:** 10.1107/S160053680900539X

**Published:** 2009-02-21

**Authors:** Mohammad Reza Melardi, Yasaman Salemi, Saba Razi Kazemi, Mohammad Kazem Rofouei

**Affiliations:** a Department of Chemistry, Islamic Azad University, Karaj Branch, Karaj, Iran; b Faculty of Chemistry, Tarbiat Moallem University, Tehran, Iran

## Abstract

In the title compound, [Hg(C_16_H_18_N_3_O_2_)Cl], the Hg^II^ atom is four-coordinated in a tetra­hedral geometry by two N atoms from the 1,3-chelating and one O atom of a 1,3-bis­(2-ethoxy­phen­yl)triazenido ligand and one terminal chloride ion. The dihedral angle between the aromatic rings is 1.72 (14)°. In the crystal C—H⋯π stacking inter­actions occur.

## Related literature

For related structures, see: Rofouei *et al.* 2008 ▶; Melardi *et al.* 2007 ▶.
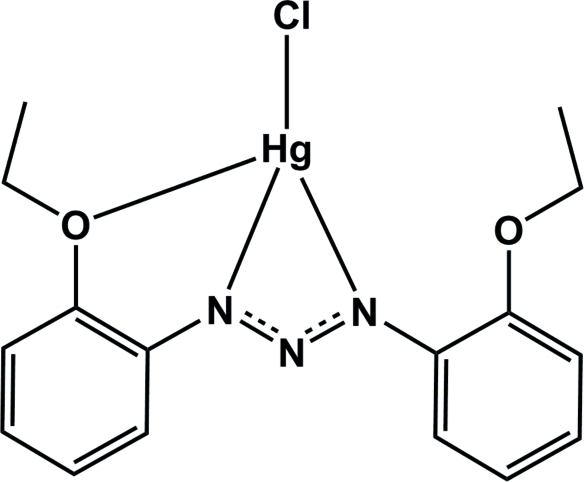


## Experimental

### 

#### Crystal data

[Hg(C_16_H_18_N_3_O_2_)Cl]
*M* *_r_* = 520.37
Monoclinic, 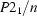

*a* = 10.1600 (5) Å
*b* = 7.3802 (4) Å
*c* = 22.5655 (11) Å
β = 97.817 (1)°
*V* = 1676.30 (15) Å^3^
*Z* = 4
Mo *K*α radiation
μ = 9.35 mm^−1^
*T* = 100 K
0.15 × 0.12 × 0.08 mm

#### Data collection

Bruker APEXII CCD area-detector diffractometer
Absorption correction: multi-scan (*APEX2*; Bruker, 2005 ▶) *T*_min_ = 0.280, *T*_max_ = 0.479
19713 measured reflections
4451 independent reflections
4009 reflections with *I* > 2σ(*I*)
*R*_int_ = 0.036

#### Refinement

*R*[*F* ^2^ > 2σ(*F* ^2^)] = 0.021
*wR*(*F* ^2^) = 0.049
*S* = 1.01
4451 reflections
210 parameters
H-atom parameters constrained
Δρ_max_ = 0.98 e Å^−3^
Δρ_min_ = −1.19 e Å^−3^

### 

Data collection: *APEX2* (Bruker, 2005 ▶); cell refinement: *APEX2*; data reduction: *SAINT* (Bruker, 2005 ▶); program(s) used to solve structure: *SHELXS97* (Sheldrick, 2008 ▶); program(s) used to refine structure: *SHELXL97* (Sheldrick, 2008 ▶); molecular graphics: *SHELXTL* (Sheldrick, 2008 ▶); software used to prepare material for publication: *SHELXTL*.

## Supplementary Material

Crystal structure: contains datablocks I, global. DOI: 10.1107/S160053680900539X/pv2138sup1.cif

Structure factors: contains datablocks I. DOI: 10.1107/S160053680900539X/pv2138Isup2.hkl

Additional supplementary materials:  crystallographic information; 3D view; checkCIF report

## Figures and Tables

**Table 1:** Hydrogen-bond geometry (Å, °)

*D*—H⋯*A*	*D*—H	H⋯*A*	*D*⋯*A*	*D*—H⋯*A*
C3—H3*A*⋯*Cg*1^i^	0.95	2.87	3.598 (3)	134
C15—H15*B*⋯*Cg*1^ii^	0.99	2.68	3.511 (3)	142

Symmetry codes: (i) 
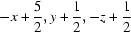
; (ii) 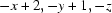. *Cg*1 is the centroid of the C1–C6 ring.

**Fig. 1.**
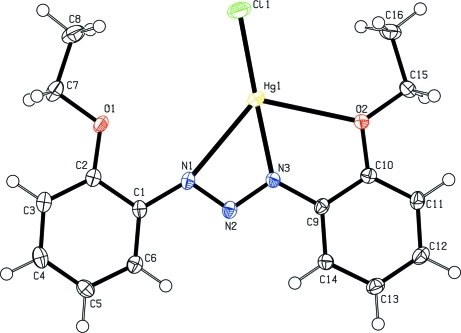
Molecular structure of the title complex. Thermal ellipsoids are drawn at 50% probability level.

**Fig. 2.**
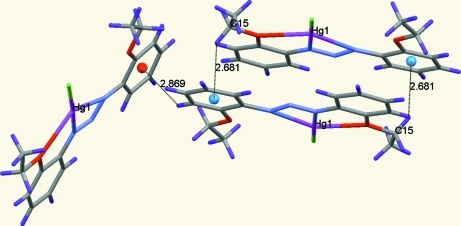
C—H···π Stacking interactions between CH groups and aromatic phenyl rings centroid.

**Fig. 3.**
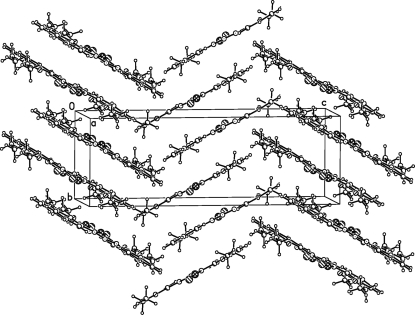
Unit cell packing diagram of the title complex.

## supplementary crystallographic information

### Comment

Recently we have reported the synthesis and crystal structure of [1,3-bis(2-methoxyphenyl)triazene with Hg^II^ as *ML*_2_ structure [Rofouei *et al.*, 2008] and [1,3-bis(2-methoxybenzene)triazene with Hg^II^ as ML structure [Melardi *et al.*, 2007]. In this article we report the synthesis and crystal structure of the title compound, (I).

In the title compound, the Hg^II^ atom is four-coordinated in a tetrahedral configuration by two N atoms from the chelating (1,3) and one O atom of ethoxyphenyl triazenido ligand and one terminal Cl atom leading to an asymmetric molecule (Fig. 1). There are interesting C—H···π stacking interactions between CH groups and aromatic phenyl rings with C—H···π distances of 2.869 Å for C3—H3A···*Cg*1 (5/2 - *x*, 1/2 + *y*, 1/2 - *z*) and 2.681 Å for C15—H15B···*Cg*1 (2 - *x*, 1 - *y*, -*z*) (*Cg*1 is centroid of C1—C6 ring) as presented in Fig. 2. The unit cell packing of the title compound showing stacking of molecules is presented at Fig. 3.

### Experimental

A solution of [1,3-bis(2-ethoxyphenyl)triazene] (1 mmol, 0.285 g) in acetonitril (10 ml) and triethylamin (0.3 ml) was added to a solution of HgCl_2_ (1 mmol, 0.271 g) in methanol (10 ml) yielded the title compound. The suitable crystals for X-ray analysis were obtained from a solution of ethyl acetate after one week. m.p. = 449-451 K.

### Refinement

All hydrogen atoms were included in the refinement at calculated positions in isotropic approximation in riding mode with distances C—H = 0.95, 0.99 and 0.98 Å for aryl, methylene and methyl groups, respectively, and *U*_iso_(H) parameters equal to 1.2*U*_eq_(C) for methylene and aryl groups and equal to 1.5*U*_eq_(C) for methyl groups.

### Crystal data

**Table:**

[Hg(C_16_H_18_N_3_O_2_)Cl]	*F*(000) = 992
*M**_r_* = 520.37	*D*_x_ = 2.062 Mg m^−^^3^
Monoclinic, *P*2_1_/*n*	Mo *K*α radiation, λ = 0.71073 Å
Hall symbol: -P 2yn	Cell parameters from 8520 reflections
*a* = 10.1600 (5) Å	θ = 3–29°
*b* = 7.3802 (4) Å	µ = 9.35 mm^−^^1^
*c* = 22.5655 (11) Å	*T* = 100 K
β = 97.817 (1)°	Prism, colorless
*V* = 1676.30 (15) Å^3^	0.15 × 0.12 × 0.08 mm
*Z* = 4	

### Data collection

**Table:**

Bruker APEXII CCD area-detector diffractometer	4451 independent reflections
Radiation source: fine-focus sealed tube	4009 reflections with *I* > 2σ(*I*)
graphite	*R*_int_ = 0.036
φ and ω scans	θ_max_ = 29.0°, θ_min_ = 1.8°
Absorption correction: multi-scan (*APEX2*; Bruker, 2005)	*h* = −13→13
*T*_min_ = 0.280, *T*_max_ = 0.479	*k* = −10→10
19713 measured reflections	*l* = −30→30

### Refinement

**Table:**

Refinement on *F*^2^	Primary atom site location: structure-invariant direct methods
Least-squares matrix: full	Secondary atom site location: difference Fourier map
*R*[*F*^2^ > 2σ(*F*^2^)] = 0.021	Hydrogen site location: inferred from neighbouring sites
*wR*(*F*^2^) = 0.049	H-atom parameters constrained
*S* = 1.01	*w* = 1/[σ^2^(*F*_o_^2^) + (0.02*P*)^2^ + 2*P*] where *P* = (*F*_o_^2^ + 2*F*_c_^2^)/3
4451 reflections	(Δ/σ)_max_ < 0.001
210 parameters	Δρ_max_ = 0.98 e Å^−^^3^
0 restraints	Δρ_min_ = −1.19 e Å^−^^3^

### Special details

**Table:**

Geometry. All e.s.d.'s (except the e.s.d. in the dihedral angle between two l.s. planes) are estimated using the full covariance matrix. The cell e.s.d.'s are taken into account individually in the estimation of e.s.d.'s in distances, angles and torsion angles; correlations between e.s.d.'s in cell parameters are only used when they are defined by crystal symmetry. An approximate (isotropic) treatment of cell e.s.d.'s is used for estimating e.s.d.'s involving l.s. planes.
Refinement. Refinement of *F*^2^ against ALL reflections. The weighted *R*-factor *wR* and goodness of fit *S* are based on *F*^2^, conventional *R*-factors *R* are based on *F*, with *F* set to zero for negative *F*^2^. The threshold expression of *F*^2^ > σ(*F*^2^) is used only for calculating *R*-factors(gt) *etc*. and is not relevant to the choice of reflections for refinement. *R*-factors based on *F*^2^ are statistically about twice as large as those based on *F*, and *R*- factors based on ALL data will be even larger.

### Fractional atomic coordinates and isotropic or equivalent isotropic displacement parameters (Å^2^)

**Table:**

	*x*	*y*	*z*	*U*_iso_*/*U*_eq_	
Hg1	0.849438 (10)	0.312052 (15)	0.054958 (5)	0.01838 (4)	
Cl1	0.65126 (8)	0.35249 (12)	0.09032 (4)	0.03193 (18)	
O1	1.0251 (2)	0.5538 (3)	0.20153 (9)	0.0187 (4)	
O2	0.82363 (19)	0.1411 (3)	−0.05286 (9)	0.0174 (4)	
N1	1.0926 (2)	0.4172 (3)	0.10284 (10)	0.0159 (4)	
N2	1.1288 (2)	0.3584 (3)	0.05434 (11)	0.0160 (5)	
N3	1.0261 (2)	0.2874 (3)	0.01919 (11)	0.0158 (5)	
C1	1.1957 (3)	0.4946 (4)	0.14348 (12)	0.0154 (5)	
C2	1.1558 (3)	0.5710 (4)	0.19570 (12)	0.0162 (5)	
C3	1.2500 (3)	0.6550 (4)	0.23737 (13)	0.0195 (6)	
H3A	1.2236	0.7097	0.2720	0.023*	
C4	1.3828 (3)	0.6591 (4)	0.22844 (13)	0.0198 (6)	
H4A	1.4464	0.7174	0.2569	0.024*	
C5	1.4232 (3)	0.5785 (4)	0.17820 (13)	0.0197 (6)	
H5A	1.5143	0.5790	0.1728	0.024*	
C6	1.3297 (3)	0.4974 (4)	0.13598 (13)	0.0175 (5)	
H6A	1.3572	0.4432	0.1015	0.021*	
C7	0.9817 (3)	0.6273 (4)	0.25429 (14)	0.0237 (6)	
H7A	0.9954	0.7601	0.2559	0.028*	
H7B	1.0324	0.5727	0.2905	0.028*	
C8	0.8362 (3)	0.5833 (5)	0.25128 (16)	0.0304 (7)	
H8A	0.8021	0.6340	0.2864	0.046*	
H8B	0.8242	0.4515	0.2507	0.046*	
H8C	0.7874	0.6359	0.2148	0.046*	
C9	1.0519 (3)	0.2108 (4)	−0.03482 (12)	0.0153 (5)	
C10	0.9451 (3)	0.1305 (4)	−0.07242 (12)	0.0148 (5)	
C11	0.9671 (3)	0.0489 (4)	−0.12541 (12)	0.0183 (5)	
H11A	0.8950	−0.0038	−0.1508	0.022*	
C12	1.0944 (3)	0.0436 (4)	−0.14161 (13)	0.0204 (6)	
H12A	1.1091	−0.0147	−0.1777	0.024*	
C13	1.1995 (3)	0.1227 (4)	−0.10547 (13)	0.0210 (6)	
H13A	1.2860	0.1197	−0.1170	0.025*	
C14	1.1789 (3)	0.2072 (4)	−0.05198 (13)	0.0183 (6)	
H14A	1.2512	0.2621	−0.0274	0.022*	
C15	0.7136 (3)	0.0536 (4)	−0.08871 (13)	0.0190 (5)	
H15A	0.7319	−0.0773	−0.0925	0.023*	
H15B	0.6993	0.1072	−0.1293	0.023*	
C16	0.5924 (3)	0.0808 (5)	−0.05819 (15)	0.0258 (6)	
H16A	0.5188	0.0086	−0.0785	0.039*	
H16B	0.5677	0.2092	−0.0598	0.039*	
H16C	0.6117	0.0423	−0.0163	0.039*	

### Atomic displacement parameters (Å^2^)

**Table:**

	*U*^11^	*U*^22^	*U*^33^	*U*^12^	*U*^13^	*U*^23^
Hg1	0.01754 (6)	0.02133 (6)	0.01728 (6)	0.00076 (4)	0.00604 (4)	−0.00146 (4)
Cl1	0.0241 (4)	0.0361 (4)	0.0390 (5)	−0.0002 (3)	0.0165 (3)	−0.0071 (3)
O1	0.0217 (10)	0.0216 (10)	0.0140 (9)	−0.0016 (8)	0.0065 (8)	−0.0038 (8)
O2	0.0142 (9)	0.0238 (10)	0.0144 (9)	−0.0025 (7)	0.0026 (8)	−0.0018 (8)
N1	0.0206 (11)	0.0148 (11)	0.0119 (10)	0.0023 (9)	0.0014 (9)	−0.0006 (9)
N2	0.0193 (11)	0.0152 (11)	0.0130 (11)	0.0024 (9)	0.0007 (9)	0.0007 (8)
N3	0.0156 (11)	0.0189 (12)	0.0127 (11)	−0.0012 (9)	0.0016 (9)	0.0002 (9)
C1	0.0193 (13)	0.0121 (12)	0.0142 (12)	0.0003 (10)	0.0000 (10)	0.0001 (10)
C2	0.0207 (13)	0.0132 (12)	0.0146 (12)	0.0013 (10)	0.0020 (10)	0.0011 (10)
C3	0.0268 (15)	0.0160 (14)	0.0150 (13)	0.0007 (11)	0.0010 (11)	−0.0007 (10)
C4	0.0259 (14)	0.0145 (13)	0.0171 (13)	−0.0006 (11)	−0.0046 (11)	0.0019 (10)
C5	0.0171 (13)	0.0196 (14)	0.0212 (14)	0.0009 (11)	−0.0017 (11)	0.0021 (11)
C6	0.0208 (13)	0.0164 (13)	0.0151 (13)	0.0027 (10)	0.0017 (11)	0.0013 (10)
C7	0.0329 (17)	0.0215 (14)	0.0182 (14)	−0.0011 (12)	0.0095 (13)	−0.0021 (11)
C8	0.0306 (17)	0.0317 (18)	0.0326 (18)	−0.0023 (14)	0.0175 (14)	−0.0018 (14)
C9	0.0179 (13)	0.0167 (13)	0.0115 (12)	0.0016 (10)	0.0023 (10)	0.0017 (10)
C10	0.0160 (12)	0.0141 (12)	0.0146 (12)	−0.0005 (10)	0.0028 (10)	0.0031 (10)
C11	0.0224 (13)	0.0196 (13)	0.0128 (12)	−0.0014 (11)	0.0016 (10)	−0.0001 (11)
C12	0.0226 (14)	0.0243 (15)	0.0150 (13)	0.0047 (11)	0.0050 (11)	0.0011 (11)
C13	0.0182 (13)	0.0262 (15)	0.0191 (14)	0.0052 (11)	0.0043 (11)	0.0004 (12)
C14	0.0161 (12)	0.0239 (15)	0.0148 (13)	−0.0010 (10)	0.0016 (10)	0.0004 (11)
C15	0.0166 (12)	0.0210 (14)	0.0186 (13)	−0.0030 (11)	−0.0006 (10)	0.0012 (11)
C16	0.0169 (13)	0.0344 (17)	0.0268 (16)	−0.0021 (12)	0.0053 (12)	0.0041 (13)

### Geometric parameters (Å, °)

**Table:**

Hg1—N3	2.074 (2)	C7—C8	1.507 (5)
Hg1—Cl1	2.2840 (8)	C7—H7A	0.9900
Hg1—N1	2.674 (2)	C7—H7B	0.9900
Hg1—O2	2.721 (2)	C8—H8A	0.9800
O1—C2	1.358 (3)	C8—H8B	0.9800
O1—C7	1.431 (3)	C8—H8C	0.9800
O2—C10	1.368 (3)	C9—C14	1.397 (4)
O2—C15	1.441 (3)	C9—C10	1.413 (4)
N1—N2	1.277 (3)	C10—C11	1.384 (4)
N1—C1	1.415 (3)	C11—C12	1.391 (4)
N2—N3	1.329 (3)	C11—H11A	0.9500
N3—C9	1.400 (4)	C12—C13	1.382 (4)
C1—C6	1.395 (4)	C12—H12A	0.9500
C1—C2	1.415 (4)	C13—C14	1.399 (4)
C2—C3	1.393 (4)	C13—H13A	0.9500
C3—C4	1.391 (4)	C14—H14A	0.9500
C3—H3A	0.9500	C15—C16	1.503 (4)
C4—C5	1.391 (4)	C15—H15A	0.9900
C4—H4A	0.9500	C15—H15B	0.9900
C5—C6	1.386 (4)	C16—H16A	0.9800
C5—H5A	0.9500	C16—H16B	0.9800
C6—H6A	0.9500	C16—H16C	0.9800
			
N3—Hg1—Cl1	176.60 (7)	C8—C7—H7B	110.3
N3—Hg1—N1	51.80 (8)	H7A—C7—H7B	108.6
Cl1—Hg1—N1	129.12 (5)	C7—C8—H8A	109.5
N3—Hg1—O2	66.09 (8)	C7—C8—H8B	109.5
Cl1—Hg1—O2	112.99 (5)	H8A—C8—H8B	109.5
N1—Hg1—O2	117.86 (6)	C7—C8—H8C	109.5
Hg1—O2—C10	109.48 (8)	H8A—C8—H8C	109.5
Hg1—O2—C15	133.07 (16)	H8B—C8—H8C	109.5
C2—O1—C7	117.4 (2)	C14—C9—N3	122.6 (3)
C10—O2—C15	117.3 (2)	C14—C9—C10	119.2 (3)
N2—N1—C1	114.7 (2)	N3—C9—C10	118.2 (2)
N2—N1—Hg1	85.01 (16)	O2—C10—C11	124.2 (2)
C1—N1—Hg1	160.18 (18)	O2—C10—C9	115.8 (2)
N1—N2—N3	110.6 (2)	C11—C10—C9	120.0 (3)
N2—N3—C9	117.0 (2)	C10—C11—C12	120.3 (3)
N2—N3—Hg1	112.58 (18)	C10—C11—H11A	119.9
C9—N3—Hg1	130.44 (19)	C12—C11—H11A	119.9
C6—C1—C2	119.4 (3)	C13—C12—C11	120.3 (3)
C6—C1—N1	125.2 (3)	C13—C12—H12A	119.9
C2—C1—N1	115.5 (2)	C11—C12—H12A	119.9
O1—C2—C3	124.6 (3)	C12—C13—C14	120.2 (3)
O1—C2—C1	116.0 (2)	C12—C13—H13A	119.9
C3—C2—C1	119.4 (3)	C14—C13—H13A	119.9
C4—C3—C2	120.2 (3)	C9—C14—C13	120.0 (3)
C4—C3—H3A	119.9	C9—C14—H14A	120.0
C2—C3—H3A	119.9	C13—C14—H14A	120.0
C3—C4—C5	120.6 (3)	O2—C15—C16	107.7 (2)
C3—C4—H4A	119.7	O2—C15—H15A	110.2
C5—C4—H4A	119.7	C16—C15—H15A	110.2
C6—C5—C4	119.6 (3)	O2—C15—H15B	110.2
C6—C5—H5A	120.2	C16—C15—H15B	110.2
C4—C5—H5A	120.2	H15A—C15—H15B	108.5
C5—C6—C1	120.8 (3)	C15—C16—H16A	109.5
C5—C6—H6A	119.6	C15—C16—H16B	109.5
C1—C6—H6A	119.6	H16A—C16—H16B	109.5
O1—C7—C8	107.0 (3)	C15—C16—H16C	109.5
O1—C7—H7A	110.3	H16A—C16—H16C	109.5
C8—C7—H7A	110.3	H16B—C16—H16C	109.5
O1—C7—H7B	110.3		
			
N3—Hg1—O2—C10	0.40 (16)	N1—C1—C2—C3	−178.1 (2)
Cl1—Hg1—O2—C10	176.84 (15)	O1—C2—C3—C4	177.3 (3)
N1—Hg1—O2—C10	−1.31 (18)	C1—C2—C3—C4	−1.8 (4)
N3—Hg1—O2—C15	175.4 (2)	C2—C3—C4—C5	−0.5 (4)
Cl1—Hg1—O2—C15	−8.2 (2)	C3—C4—C5—C6	1.6 (4)
N1—Hg1—O2—C15	173.7 (2)	C4—C5—C6—C1	−0.5 (4)
N3—Hg1—N1—N2	1.55 (15)	C2—C1—C6—C5	−1.8 (4)
Cl1—Hg1—N1—N2	−174.26 (12)	N1—C1—C6—C5	179.3 (3)
O2—Hg1—N1—N2	3.54 (17)	C2—O1—C7—C8	−177.5 (2)
N3—Hg1—N1—C1	177.1 (6)	N2—N3—C9—C14	0.1 (4)
Cl1—Hg1—N1—C1	1.3 (6)	Hg1—N3—C9—C14	179.6 (2)
O2—Hg1—N1—C1	179.1 (5)	N2—N3—C9—C10	178.6 (2)
C1—N1—N2—N3	179.6 (2)	Hg1—N3—C9—C10	−2.0 (4)
Hg1—N1—N2—N3	−2.03 (19)	C15—O2—C10—C11	3.1 (4)
N1—N2—N3—C9	−177.6 (2)	C15—O2—C10—C9	−177.3 (2)
N1—N2—N3—Hg1	2.8 (3)	C14—C9—C10—O2	−179.4 (2)
N1—Hg1—N3—N2	−1.61 (15)	N3—C9—C10—O2	2.1 (4)
N1—Hg1—N3—C9	178.9 (3)	C14—C9—C10—C11	0.3 (4)
N2—N1—C1—C6	−5.0 (4)	N3—C9—C10—C11	−178.2 (2)
Hg1—N1—C1—C6	179.8 (4)	O2—C10—C11—C12	−179.6 (3)
N2—N1—C1—C2	176.1 (2)	C9—C10—C11—C12	0.7 (4)
Hg1—N1—C1—C2	0.9 (7)	C10—C11—C12—C13	−1.2 (4)
C7—O1—C2—C3	0.0 (4)	C11—C12—C13—C14	0.6 (5)
C7—O1—C2—C1	179.1 (2)	N3—C9—C14—C13	177.6 (3)
C6—C1—C2—O1	−176.3 (2)	C10—C9—C14—C13	−0.9 (4)
N1—C1—C2—O1	2.7 (4)	C12—C13—C14—C9	0.4 (4)
C6—C1—C2—C3	2.9 (4)	C10—O2—C15—C16	179.2 (2)

### Hydrogen-bond geometry (Å, °)

**Table:**

*D*—H···*A*	*D*—H	H···*A*	*D*···*A*	*D*—H···*A*
C3—H3A···Cg1^i^	0.95	2.87	3.598 (3)	134
C15—H15B···Cg1^ii^	0.99	2.68	3.511 (3)	142

Symmetry codes: (i) −*x*+5/2, *y*+1/2, −*z*+1/2; (ii) −*x*+2, −*y*+1, −*z*.
